# Editorial: Sex differences and cardiovascular therapeutics

**DOI:** 10.3389/fcvm.2024.1420293

**Published:** 2024-05-20

**Authors:** Fatma Saaoud, Keman Xu, Yifan Lu, Ying Shao, Xiaohua Jiang, Hong Wang, Xiaofeng Yang

**Affiliations:** ^1^ Lemore Center for Integrated Lymphatics and Vascular Research, Lewis Katz School of Medicine at Temple University, Philadelphia, PA, United States; ^2^ Centers of Metabolic Disease Research and Thrombosis Research Center, Departments of Cardiovascular Sciences, Lewis Katz School of Medicine at Temple University, Philadelphia, PA, United States

**Keywords:** cardiovascular diseases, sex difference, sex hormones, genetic factors, therapeutics

**Editorial on the Research Topic** Sex differences and cardiovascular therapeutics

## Introduction

Cardiovascular diseases (CVDs) remain a leading cause of morbidity and mortality worldwide (1). Sex and gender as a biological variable, are crucial factors that impact every aspect of clinical and public health practice and research (2–4). In recent times, research investigating sex differences has gained more attention, partly benefit from federal agencies emphasizing the importance of considering sex as a significant biological factor (5). Within cardiovascular medicine, sex and gender affect disease presentation, pathophysiology, diagnostic assessment, responses to treatments, and overall health outcomes (6, 7). Historically, CVD has been perceived as primarily affecting men; however, it is increasingly recognized as a leading cause of morbidity and mortality in women as well (8). While men tend to develop CVD earlier in life, CVD prevalence increases significantly in postmenopausal women, narrowing the gap between the sexes (9). Men have traditionally experienced higher rates of CVD-related events, but women are more likely to die following an acute cardiovascular event (10). The existence of these gender disparities has prompted significant attention, highlighting the crucial significance of considering gender variations in the prevention, diagnosis, treatment, and overall management of CVD (11).

Traditional risk factors such as hypertension, diabetes, dyslipidemia, and smoking affect both sexes, but their impacts can vary between men and women (12–14). Additionally, women may experience unique risk factors including pregnancy-related complications such as gestational diabetes and preeclampsia, as well as endocrine disorders in reproductive age such as polycystic ovary syndrome (PCOS) and early menopause, which are associated with accelerated development of CVD and impaired CVD-free survival (15–17). Biological differences include genetic differences, variation in sex hormonal status, vascular anatomy, endothelial function, and plaque composition, which contribute to differences in the pathophysiology of CVD between men and women (18, 19). Women showed less plaque inflammatory infiltration compared to plaques from age-matched men (20–22). In addition, women often undergo fewer diagnostic tests and experience delays in diagnosis compared to men, leading to disparities in timely intervention and treatment (23). Despite accumulating evidence, the precise roles of biological sex and the sociocultural aspect of gender in the development and consequences of CVDs have not been fully explained. The interplay between sex-specific disparities in genetic and hormonal mechanisms and the intricate nature of gender, including its various components and influencing factors, which give rise to different disease patterns in men and women, requires further investigation.

The extents to which biological factors, such as genes and hormones, contribute to cardiovascular traits and outcomes are still not fully grasped. Heightened recognition of gender's impact has prompted endeavors to assess gender in both retrospective and prospective clinical studies, leading to the creation of gender scores. Yet, the combined or conflicting influences of sex and gender on cardiovascular characteristics, as well as on the mechanisms underlying CVDs, have not been systematically elucidated. The majority of medication are withdrawn after FDA approval due to unexpected adverse effects in women (24). Additionally, there are differences in the effectiveness and side effects of cardiovascular medications between men and women (25). Current guidelines do not provide sex-specific recommendations on the use of antithrombotic drugs in patients with coronary artery disease. Nevertheless, the effectiveness of antithrombotic medications might be impacted by genetic and biological factors associated to sex (26). Women generally exhibit greater platelet reactivity at baseline and in response to low-dose aspirin treatment in comparison to men (27). Despite receiving high-dose statin therapy following acute coronary syndrome, women showed a smaller absolute reduction in low-density lipoprotein cholesterol (LDL) cholesterol levels compared to men (28). Understanding these sex differences is crucial for providing personalized and effective cardiovascular care. It requires including more women in clinical trials, analyzing data by sex, and considering sex-specific factors in treatment decisions. By doing so, healthcare providers can optimize outcomes and reduce disparities in cardiovascular care between men and women.

## Sex hormones in CVD

Sex hormones, including estrogen, progesterone, and testosterone, play significant roles in cardiovascular health and disease (29, 30). Estrogen, primarily found in premenopausal women, exerts cardioprotective effects (8, 29, 31). It helps maintain healthy blood vessel function by promoting vasodilation, reducing inflammation, and inhibiting the formation of atherosclerotic plaques. Estrogen also influences lipid metabolism, favoring higher levels of high-density lipoprotein (HDL) cholesterol and lower levels of LDL cholesterol, contributing to a reduced risk of CVDs such as heart attacks and strokes (32, 33).

The differences in estrogen levels between men and women, as well as the changes that occur during menopause, contribute to the variation in CVD occurrence between the sexes. Before menopause, women generally have higher levels of estrogen, potentially explaining their lower risk of CVD compared to men of the same age. However, after menopause, when estrogen levels decline, women's risk of CVD increases and may approach that of men (34). Progesterone, another female sex hormone, also plays a role in cardiovascular health, though its effects are less well understood compared to estrogen. Some research suggests that progesterone may have protective effects on the cardiovascular system, such as promoting vasodilation and inhibiting smooth muscle cell proliferation in blood vessels (35, 36).

Androgens, including testosterone and other male sex hormones, can influence cardiovascular health in both men and women. Low levels of testosterone in men have been associated with an increased risk of CVD, including coronary artery disease and heart failure (18). Testosterone influences factors such as blood pressure regulation, lipid metabolism, and the development of atherosclerosis. However, the relationship between testosterone levels and cardiovascular risk is complex, and both low and high levels of testosterone have been implicated in various cardiovascular conditions (33). In addition to testosterone, other androgens such as dehydroepiandrosterone (DHEA) and its sulfate (DHEAS) may also impact cardiovascular risk factors (18).

Overall, sex hormones play intricate roles in cardiovascular physiology and pathology. Understanding the interplay between sex hormones and cardiovascular health is essential for developing personalized approaches to preventing and managing CVDs.

## Genetic factors that are specific to each sex and influence cardiovascular characteristics

Genetic factors play a significant role in cardiovascular characteristics, with some of these factors being specific to each sex (37). The presence of sex chromosomes (XX in females and XY in males) not only determines primary sexual characteristics but also influences cardiovascular health (38, 39). For instance, genes on the Y chromosome may impact cardiac function (40). Moreover, genetic variations in lipoprotein metabolism can influence the metabolism of lipoproteins differently in men and women (41, 42). For example, certain genetic variants may have a more pronounced effect on the levels of HDL cholesterol in women compared to men, or vice versa (43). Additionally, genes involved in blood pressure regulation may exhibit sex-specific effects. For instance, variations in genes related to the renin-angiotensin-aldosterone system (RAAS) may influence blood pressure in varying ways between men and women (44). Understanding these sex-specific genetic factors is crucial for developing personalized approaches to cardiovascular disease prevention, diagnosis, and treatment. It underscores the importance of considering sex as a biological variable in cardiovascular research and clinical practice.

Our Research Topic: “*Sex Differences and Cardiovascular Therapeutics*,” featured twelve papers comprising original research papers and reviews (Table 1). These highlights offer a comprehensive perspective on gender-related differences in various CVDs, potential factors contributing to these distinctions, and management strategies. Collectively, these papers contribute to our understanding of sex differences in cardiovascular therapeutics and emphasize the importance of tailored approaches to prevention, diagnosis, and treatment based on gender-specific considerations.

**Table 1:** Twelve highly viewed research papers, published in our special topic entitled “*Sex differences and cardiovascular therapeutics*”, are summarized.

Paper title	Summary	References
Sex differences in the renin-angiotensin aldosterone system and its roles in hypertension, cardiovascular, and kidney diseases	- Hypertension is less common in premenopausal women than in men. 1. Animal studies have demonstrated that females have greater nitric oxide (NO) bioavailability than males due to a higher capacity for generating NO in women, while increased oxidative stress in men leads to endothelial dysfunction and activation of the renin-angiotensin-aldosterone system (RAAS). 2. The RAAS is regulated by estrogen, which binds to estrogen receptor-α (ER-α) expressed in the vascular endothelium, promoting endothelial repair, vasodilation, and NO production. 3. Estrogen can modify RAAS activity by controlling the expression of key substrates, enzymes, receptors, and protein synthesis. 4. Estrogen has antihypertensive effects by upregulating substrates and enzymes in counter-regulatory RAAS pathways, such as increased expression of ACE2 and activation of the MAS receptor and Ang III/AT2 receptor. - Compared to men, women with high blood pressure had a higher risk for adverse cardiac events. - Women had worse blood pressure control rates compared to men (SPRINT trial). - There is an increased prevalence of adverse drug reactions in females due to increased drug bioavailability.	Nwia et al.
Sex differences in patterns of referral and resource utilization in the cardiology clinic: an outpatient analysis	There were higher referral rates of women compared to men from primary care due to palpitations in women (n = 676; 19.2%) and ECG abnormalities in men (n = 570; 23.2%). Additionally, compared to men, women were older. Women also had fewer cardiology hospitalizations and a lower mortality rate. Moreover, women under 65 years old had more admission to the emergency rooms.	Vicent et al.
Association of sex with post-arrest care and outcomes after out-of-hospital cardiac arrest of initial shockable rhythm: a nationwide cohort study	Compared to men, women were older. There were no significant differences observed in survival outcomes between males and females. Additionally, there was no significant difference noted between male and female patients who received in-hospital interventions such as extracorporeal cardiopulmonary resuscitation or targeted temperature management.	Hosomi et al.
Sex-specific association of low-renin hypertension with metabolic and musculoskeletal health in Korean older adults	In postmenopausal women, low-renin hypertension was associated to a lower femur neck T-score and a deteriorated trabecular bone score, indicating an increased risk of osteoporosis and subsequent fracture.	Lee et al.
Oxidative stress and inflammation distinctly drive molecular mechanisms of diastolic dysfunction and remodeling in female and male heart failure with preserved ejection fraction rats	Ren-2 male transgenic (TG) rats exhibited cardiac enlargement, left ventricular hypertrophy, left ventricle diastolic dysfunction, and hypertension compared to female TG rats. Both males and females displayed high levels of proinflammatory cytokines, along with significant alterations in apoptotic and autophagy pathways.	Zhazykbayeva et al.
Sex differences in long QT syndrome	There is a higher incidence of Long QT Syndrome (LQTS), with malignant arrhythmias being associated with female sex during postpartum, menopausal, and perimenopausal due to sex hormone differences. Women with LQTS show a higher risk of ventricular tachycardia and sudden cardiac death in comparison to the relatively low risk during pregnancy.	Díez-Escuté et al.
Diabetes and heart failure associations in women and men Results from the MORGAM consortium	Men have a greater absolute risk of heart failure than women regardless of diabetes status. Additionally, there are no sex-specific differences in the relative risk of heart failure between diabetic men and women.	Chadalavada et al.
The role of the pregnancy heart team in clinical practice	There are increased maternal mortality and morbidity rates in pregnant women with cardiovascular diseases. Peripartum cardiomyopathy and pre-existing cardiovascular diseases are the leading causes of heart failure during pregnancy. The prevalence of acute myocardial infarction associated with pregnancy also increases during pregnancy. Furthermore, there is an increased risk of corrected congenital heart disease, valvular heart diseases, and cardiomyopathies during pregnancy.	Lucà et al.
The emerging role of estrogen's non-nuclear signaling in the cardiovascular disease	Estrogen protects against vascular injury, suppress neointima hyperplasia, and reduces the development of atherosclerosis. Additionally, estrogen suppresses metabolic disorders and reduces pressure overload-induced cardiac hypertrophy.	Tokiwa et al.
Sex differences in coronary artery bypass grafting-related morbidity and mortality	CABG in women exhibited a stronger correlation with elevated risks of diseases such as hypertension, type 1 diabetes, diabetic retinopathy, Alzheimer's disease, aortic aneurysms, gout, and chronic kidney disease compared to the risk increases noted in men. Additionally, there was an increased risk of cardiac death after CABG in women compared to men.	Nurkkala et al.
Sex difference and outcome trends following transcatheter aortic valve replacement	Men exhibited markedly higher prevalence rates of hyperlipidemia, diabetes mellitus, chronic renal disease, peripheral artery disease, and coronary artery disease. Men showed a higher prevalence of previous cardiac interventions, including prior percutaneous coronary intervention, and a greater frequency of prior device implantation. Women demonstrated elevated in-hospital mortality rates. Additionally, women exhibited significantly higher rates of pericardial, cardiac, pulmonary, hemorrhagic, vascular, and neurological complications. Conversely, men had higher rates of acute renal failure, device-related mechanical complications, and pacemaker implantation.	Elbaz-Greener et al.
Cardiovascular sex-differences: insights via physiology-based modeling and potential for noninvasive sensing via ballistocardiography	On average, the size of the female heart is approximately one-fourth smaller than that of the male heart. In women, the Left Ventricle (LV) is typically smaller than in men, resulting in lower end-diastolic volume (EDV) and end-systolic volume (ESV), as well as smaller stroke volume (SV). LV ejection fraction (EF) is higher in women than in men. Additionally, women exhibit a smaller size and higher contractility of the Right Ventricle (RV), leading to lower EDV, SV, ESV, and cardiac output (CO) compared to men. Vessel diameters and lengths tend to be smaller in females when compared to males.	Zaid et al.

## Conclusion

Sex differences play a significant role in the epidemiology, pathophysiology, clinical presentation, diagnosis, management, and outcomes of cardiovascular diseases. Recognizing these differences and implementing sex-specific approaches in research, clinical practice, and public health initiatives are essential for reducing disparities and improving cardiovascular outcomes for both men and women. While the differences between sexes in the occurrence and complications of CVDs are widely acknowledged, there are relatively limited data in both clinical and pre-clinical studies that adequately explore the underlying mechanisms regarding sex as a biological variable in CVDs.

## References

[1] Roth G Abat D Abat K Abay S Abbafati C Abbasi N Global, regional, and national age-sex-specific mortality for 282 causes of death in 195 countries and territories, 1980–2017: a systematic analysis for the global burden of disease study 2017. Lancet. (2018) 392(10159):1736–88. 10.1016/S0140-6736(18)32203-7 30496103 PMC6227606

[2] Clayton JA Tannenbaum C. Reporting sex, gender, or both in clinical research? JAMA. (2016) 316(18):1863–4. 10.1001/jama.2016.16405 27802482

[3] Schiebinger L Stefanick ML. Gender matters in biological research and medical practice. J Am Coll Cardiol. (2016) 67(2):136–8. 10.1016/j.jacc.2015.11.029 26791058

[4] Mauvais-Jarvis F Bairey Merz N Barnes P Brinton R Carrero JJ DeMeo D Sex and gender: modifiers of health, disease, and medicine. Lancet. (2020) 396(10250):565–82. 10.1016/S0140-6736(20)31561-0 32828189 PMC7440877

[5] Clayton JA. Applying the new SABV (sex as a biological variable) policy to research and clinical care. Physiol Behav. (2018) 187:2–5. 10.1016/j.physbeh.2017.08.012 28823546

[6] Spence JD Pilote L. Importance of sex and gender in atherosclerosis and cardiovascular disease. Atherosclerosis. (2015) 241(1):208–10. 10.1016/j.atherosclerosis.2015.04.806 25980844

[7] Miller L Marks C Becker J Hurn P Chen WJ Woodruff T Considering sex as a biological variable in preclinical research. Faseb J. (2017) 31(1):29–34. 10.1096/fj.201600781r 27682203 PMC6191005

[8] Connelly PJ Jandeleit-Dahm KAM Delles C. Sex and gender aspects in vascular pathophysiology. Clin Sci (Lond). (2020) 134(16):2203–7. 10.1042/CS20200876 32844996

[9] den Ruijter HM Haitjema S Asselbergs FW Pasterkamp G. Sex matters to the heart: a special issue dedicated to the impact of sex related differences of cardiovascular diseases. Atherosclerosis. (2015) 241(1):205–7. 10.1016/j.atherosclerosis.2015.05.003 26003338

[10] Yu Y Chen J Li D Wang L Wang W Liu H. Systematic analysis of adverse event reports for sex differences in adverse drug events. Sci Rep. (2016) 6: 24955. 10.1038/srep24955 27102014 PMC4840306

[11] Seeland U Regitz-Zagrosek V. Sex and gender differences in cardiovascular drug therapy. Handb Exp Pharmacol. (2012) 214:211–36. 10.1007/978-3-642-30726-3_11 23027453

[12] Dawber TR Moore FE Mann GV. Coronary heart disease in the framingham study. Am J Public Health Nations Health. (1957) 47(4 Pt 2):4–24. 10.2105/AJPH.47.4_Pt_2.4 13411327 PMC1550985

[13] Mahmood SS Levy D Vasan RS Wang TJ. The Framingham heart study and the epidemiology of cardiovascular disease: a historical perspective. Lancet. (2014) 383(9921):999–1008. 10.1016/S0140-6736(13)61752-3 24084292 PMC4159698

[14] Feldman RD. Sex-specific determinants of coronary artery disease and atherosclerotic risk factors: estrogen and beyond. Can J Cardiol. (2020) 36(5):706–11. 10.1016/j.cjca.2020.03.002 32389343

[15] Veltman-Verhulst SM van Rijn BB Westerveld HE Franx A Bruinse HW Fauser BC Polycystic ovary syndrome and early-onset preeclampsia: reproductive manifestations of increased cardiovascular risk. Menopause. (2010) 17(5):990–6. 10.1097/gme.0b013e3181ddf705 20551845

[16] Sattar N Greer IA. Pregnancy complications and maternal cardiovascular risk: opportunities for intervention and screening? Br Med J. (2002) 325(7356):157–60. 10.1136/bmj.325.7356.157 12130616 PMC1123678

[17] Ray JG Vermeulen MJ Schull MJ Redelmeier DA. Cardiovascular health after maternal placental syndromes (CHAMPS): population-based retrospective cohort study. Lancet. (2005) 366(9499):1797–803. 10.1016/S0140-6736(05)67726-4 16298217

[18] Zhao D Guallar E Ballantyne CM Post WS Ouyang P Vaidya D Sex hormones and incident heart failure in men and postmenopausal women: the atherosclerosis risk in communities study. J Clin Endocrinol Metab. (2020) 105(10):e3798–807. 10.1210/clinem/dgaa500 32770207 PMC7455306

[19] Ventura-Clapier R Piquereau J Garnier A Mericskay M Lemaire C Crozatier B. Gender issues in cardiovascular diseases. Focus on energy metabolism. Biochim Biophys Acta Mol Basis Dis. (2020) 1866(6):165722. 10.1016/j.bbadis.2020.165722 32057941

[20] Wendorff C Wendorff H Pelisek J Tsantilas P Zimmermann A Zernecke A Carotid plaque morphology is significantly associated with sex, age, and history of neurological symptoms. Stroke. (2015) 46(11):3213–9. 10.1161/STROKEAHA.115.010558 26451032

[21] Sangiorgi G Roversi S Biondi Zoccai G Modena MG Servadei F Ippoliti A Sex-related differences in carotid plaque features and inflammation. J Vasc Surg. (2013) 57(2):338–44. 10.1016/j.jvs.2012.07.052 23058720

[22] Yuan XM Ward LJ Forssell C Siraj N Li W. Carotid atheroma from men has significantly higher levels of inflammation and iron metabolism enabled by macrophages. Stroke. (2018) 49(2):419–25. 10.1161/STROKEAHA.117.018724 29284736

[23] Bugiardini R Ricci B Cenko E Vasiljevic Z Kedev S Davidovic G Delayed care and mortality among women and men with myocardial infarction. J Am Heart Assoc. (2017) 6(8):e005968. 10.1161/JAHA.117.005968 PMC5586439 28862963

[24] Domecq C Naranjo CA Ruiz I Busto U. Sex-related variations in the frequency and characteristics of adverse drug reactions. Int J Clin Pharmacol Ther Toxicol. (1980) 18(8):362–6. 7409941

[25] Bairey Merz CN Mark S Boyan BD Jacobs AK Shah PK Shaw LJ Proceedings from the scientific symposium: sex differences in cardiovascular disease and implications for therapies. J Womens Health (Larchmt). (2010) 19(6):1059–72. 10.1089/jwh.2009.1695 20500123 PMC2940456

[26] Levin RI. The puzzle of aspirin and sex. N Engl J Med. (2005) 352(13):1366–8. 10.1056/NEJMe058051 15755763

[27] Price MJ Nayak KR Barker CM Kandzari DE Teirstein PS. Predictors of heightened platelet reactivity despite dual-antiplatelet therapy in patients undergoing percutaneous coronary intervention. Am J Cardiol. (2009) 103(10):1339–43. 10.1016/j.amjcard.2009.01.341 19427425

[28] Truong QA Murphy SA McCabe CH Armani A Cannon CP, TIMI Study Group. Benefit of intensive statin therapy in women: results from PROVE IT-TIMI 22. Circ Cardiovasc Qual Outcomes. (2011) 4(3):328–36. 10.1161/CIRCOUTCOMES.110.957720 21487089 PMC3097284

[29] Clegg D Hevener AL Moreau KL Morselli E Criollo A Van Pelt RE Sex hormones and cardiometabolic health: role of estrogen and estrogen receptors. Endocrinology. (2017) 158(5):1095–105. 10.1210/en.2016-1677 28323912 PMC6283431

[30] Morselli E Santos RS Criollo A Nelson MD Palmer BF Clegg DJ. The effects of oestrogens and their receptors on cardiometabolic health. Nat Rev Endocrinol. (2017) 13(6):352–64. 10.1038/nrendo.2017.12 28304393

[31] Regitz-Zagrosek V Kararigas G. Mechanistic pathways of sex differences in cardiovascular disease. Physiol Rev. (2017) 97(1):1–37. 10.1152/physrev.00021.2015 27807199

[32] Pabbidi MR Kuppusamy M Didion SP Sanapureddy P Reed JT Sontakke SP. Sex differences in the vascular function and related mechanisms: role of 17β-estradiol. Am J Physiol Heart Circ Physiol. (2018) 315(6):H1499–518. 10.1152/ajpheart.00194.2018 30192631

[33] dos Santos RL da Silva FB Ribeiro RF Jr Stefanon I. Sex hormones in the cardiovascular system. Horm Mol Biol Clin Investig. (2014) 18(2):89–103. 10.1515/hmbci-2013-0048 25390005

[34] Garcia M Mulvagh SL Merz CN Buring JE Manson JE. Cardiovascular disease in women: clinical perspectives. Circ Res. (2016) 118(8):1273–93. 10.1161/CIRCRESAHA.116.307547 27081110 PMC4834856

[35] Bernstein P Pohost G. Progesterone, progestins, and the heart. Rev Cardiovasc Med. (2010) 11(3):e141–9. 10.3909/ricm0557 21045766

[36] Prior JC Elliott TG Norman E Stajic V Hitchcock CL. Progesterone therapy, endothelial function and cardiovascular risk factors: a 3-month randomized, placebo-controlled trial in healthy early postmenopausal women. PLoS One. (2014) 9(1):e84698. 10.1371/journal.pone.0084698 24465425 PMC3897380

[37] Hajar R. Genetics in cardiovascular disease. Heart Views. (2020) 21(1):55–6. 10.4103/HEARTVIEWS.HEARTVIEWS_140_19 32082505 PMC7006335

[38] Abramowitz LK Olivier-Van Stichelen S Hanover JA. Chromosome imbalance as a driver of sex disparity in disease. J Genomics. (2014) 2:77–88. 10.7150/jgen.8123 25031659 PMC4091450

[39] Reue K Wiese CB. Illuminating the mechanisms underlying sex differences in cardiovascular disease. Circ Res. (2022) 130(12):1747–62. 10.1161/CIRCRESAHA.122.320259 35679362 PMC9202078

[40] Praktiknjo SD Picard S Deschepper CF. Comparisons of chromosome Y-substituted mouse strains reveal that the male-specific chromosome modulates the effects of androgens on cardiac functions. Biol Sex Differ. (2016) 7:61. 10.1186/s13293-016-0116-4 27980711 PMC5143463

[41] Holven KB van Lennep JR. Sex differences in lipids: a life course approach. Atherosclerosis. (2023) 384:117270. 10.1016/j.atherosclerosis.2023.117270 37730457

[42] Tabassum R Widén E Ripatti S. Effect of biological sex on human circulating lipidome: an overview of the literature. Atherosclerosis. (2023) 384:117274. 10.1016/j.atherosclerosis.2023.117274 37743161

[43] Link JC Chen X Prien C Borja MS Hammerson B Oda MN Increased high-density lipoprotein cholesterol levels in mice with XX versus XY sex chromosomes. Arterioscler Thromb Vasc Biol. (2015) 35(8):1778–86. 10.1161/ATVBAHA.115.305460 26112012 PMC4668127

[44] Sampson AK Andrews KL Graham D McBride MW Head GA Thomas MC Origin of the Y chromosome influences intrarenal vascular responsiveness to angiotensin I and angiotensin (1–7) in stroke-prone spontaneously hypertensive rats. Hypertension. (2014) 64(6):1376–83. 10.1161/HYPERTENSIONAHA.114.03756 25201895

